# Multimethod latent class analysis

**DOI:** 10.3389/fpsyg.2015.01332

**Published:** 2015-09-17

**Authors:** Fridtjof W. Nussbeck, Michael Eid

**Affiliations:** ^1^Department of Psychology, Bielefeld UniversityBielefeld, Germany; ^2^Department of Education and Psychology, Freie Universitaet BerlinBerlin, Germany

**Keywords:** latent-class analysis, rater agreement, MTMM-analysis, log-linear modeling, rater bias

## Abstract

Correct and, hence, valid classifications of individuals are of high importance in the social sciences as these classifications are the basis for diagnoses and/or the assignment to a treatment. The via regia to inspect the validity of psychological ratings is the multitrait-multimethod (MTMM) approach. First, a latent variable model for the analysis of rater agreement (latent rater agreement model) will be presented that allows for the analysis of convergent validity between different measurement approaches (e.g., raters). Models of rater agreement are transferred to the level of latent variables. Second, the latent rater agreement model will be extended to a more informative MTMM latent class model. This model allows for estimating (i) the convergence of ratings, (ii) method biases in terms of differential latent distributions of raters and differential associations of categorizations within raters (specific rater bias), and (iii) the distinguishability of categories indicating if categories are satisfyingly distinct from each other. Finally, an empirical application is presented to exemplify the interpretation of the MTMM latent class model.

## Introduction

In many areas of the social and behavioral sciences, researchers as well as practitioners have to classify individuals according to some predefined categories. Examples are the categories of a rating scale measuring a personality trait such as neuroticism, extraversion, or conscientiousness. Another example is a clinical classification system that consists of categories representing different syndromes. Burns and Haynes (2006) emphasize the great importance of having valid classification systems that are based on psychological or clinical judgments. Invalid categorizations may lead to wrong diagnoses or the assignment to an inadequate treatment.

One of the most prominent approaches to examine the validity of psychological measures is the multitrait-multimethod (MTMM) analysis proposed by Campbell and Fiske (1959). Their original approach was the starting point for the development of a great number of different MTMM analysis strategies (see e.g., Eid et al., 2006). Most of the MTMM approaches proposed so far require continuous or at least ordinal observed variables. Latent variable approaches that are developed in this context assume that the latent variables are continuous. However, many constructs (latent variables) are not continuous in nature but are categorical. Clinical disorders, personality types, or attribution-styles may better be represented using non-ordered categories than continuous latent variables. To date no latent variable models exist that can be used to inspect the convergent and discriminant validity of non-ordered categories. Such a model would bear the advantages that multiple indicators for every construct could be used instead of one broad categorization reflecting the construct. Hence, categorizations could rely on more easily detectable overt behavior (being self-doubtful) instead of the more abstract psychological construct (being neurotic), measurement error affecting manifest ratings (raters may hesitate to choose one out of two categories) could be controlled for inspecting associations at the latent level, differences between the administered methods (e.g., raters) could be revealed inspecting the associations between the latent categories and the manifest categories, and item specificities (items covering more or less frequent behaviors) can be considered. Therefore, the model would be able to separate unsystematic measurement error from systematic method-specific effects. We will present a multimethod latent class model that fills this gap. We will first shortly review existing indices and models of rater agreement that can be used to analyze the convergence of different methods. Then, we will show how these approaches can be extended to a more informative MTMM model.

## Indices and models of rater agreement

A first approach to investigate the convergent validity of non-ordered categorical ratings (measures) is analyzing the agreement between different raters (or other kinds of methods). In order to investigate the agreement between different raters, the two raters must use the same categories in classifying individuals. The standard approach focuses on agreement either as overall agreement or as category specific agreement. Examples of overall agreement indices are Cohen's kappa, the proportion agreement index as well as occurrence- and non-occurrence indices (Agresti, 1992, 2013; Nussbeck, 2006). Approaches that focus on category-specific agreement typically are defined within the framework of log-linear modeling. Log-linear models focus on the cell-frequencies in cross-classifications of two or more variables; that is, they focus not only on cells on the main diagonal representing agreement (as for agreement indices) but also on cells besides the main diagonal representing disagreement between methods (raters).

Using overall agreement indices bears the advantage that only one coefficient has to be calculated representing the “average” or “overall” agreement. However, although these indices provide information about the amount of agreement, they do not provide differential information about category-specific agreement or information about the sources of disagreement. Yet, it is important to know, whether agreement or disagreement is especially frequent for some category combinations or whether it is a general property of the two methods. This information can only be retrieved by log-linear models. Log-linear rater agreement models are tailored to reflect patterns of agreement (as a constant agreement rate or category-specific agreement rates) and disagreement (no systematic pattern of disagreement or systematic patterns of disagreement) imposing meaningful restrictions on model parameters (Agresti, 1992; Nussbeck, 2006). At the level of manifest variables, four different rater agreement models have been developed: (i) the quasi-independence model I, (ii) the quasi-independence model II, (iii) the quasi-symmetry model, and (iv) the symmetry model (for a more detailed discussion see e.g., Agresti, 1992; Nussbeck, 2006).

However, these models and the previously mentioned agreement indices suffer from one major limitation. They do not allow for the analysis of more than one construct measured by one indicator per method (rater). Assuming that rater agreement depends on the categories of the items administered (it is harder to judge if somebody is moody than if this person is self-doubtful, although both adjectives are used to measure neuroticism, for example), the construct (some may be more easily detectable, e.g., sociability vs. neuroticism), and the raters (peers may be better raters than acquaintances), it is necessary to extend the existing models to more indicators and to more traits.

Extending rater agreement models to models with multiple indicators per trait-method (rater) unit would allow for identifying the categories of an underlying latent variable (so called latent classes, types, or statuses) on which the different observed response patterns (observed scores on the multiple indicators) depend. Multiple indicators are necessary because many symptoms or trait categories can *not* be directly observed (e.g., psychiatric syndromes and disorders) but have to be deduced relying on multiple observations (which themselves may be classifications of overt behaviors). If, for example, a researcher is interested in the adequacy of psychiatric diagnoses of different raters relying on the DSM-V (American Psychiatric Association, 2013) it may be worthwhile not only to examine the final classification but also to inspect the ratings of the single check-list categories. This inspection can reveal if (a) all raters agree with respect to the check-list categories, (b) if they come to the same conclusions about the status of the patient, (c) if all categories are weighted to the same degree across raters to produce the final diagnoses, and (d) if the categories of the observed variables represent the latent variables. Latent rater agreement models could allow for a detailed analysis of the agreement and disagreement on the level of latent categories. Integrating additional constructs by considering multiple traits would allow for an analysis if there is higher or lower agreement for particular constructs and how the different categories of the different latent variables co-occur yielding information about discriminant validity. Moreover, these models would allow to disentangle unsystematic measurement error from systematic method-specific influences.

## Latent rater agreement models

The extension of log-linear rater agreement models to rater agreement models with latent variables will be done in two steps. In a first step, log-linear models with latent variables will be introduced and their reformulation as latent class models will be presented. In a second step, the structure of the rater agreement models will be defined at the level of latent variables.

The starting point of log-linear models with latent variables (Goodman, 1974a,b; Habermann, 1979; Hagenaars, 1990, 1993; Vermunt, 1997b) is a multi-way frequency table cross-classifying all observables and latent categories. The frequencies of the latent (not directly observable) categories can be estimated relying on maximum likelihood procedures (see e.g., Habermann, 1979; Vermunt, 1997a). Equation (1) depicts the effect-coded standard log-linear latent class model (LCM) for the example of four manifest indicators *A, B, C*, and *D* and one latent variable *W*:

(1) $$\begin{array}{l} e_{abcdw}=\eta \tau_{w}^{W}\tau_{a}^{A}\tau_{b}^{B}\tau_{c}^{C}\tau_{d}^{D}\tau_{aw}^{AW}\tau_{bw}^{BW}\tau_{cw}^{CW}\tau_{dw}^{DW}. \end{array}$$

The expected frequency (*e*_*abcdw*_) of a particular cell in the multi-way frequency table is the product of different parameters. The parameter η is the overall geometric mean of the expected frequencies of all cells. The τ-parameters (with the name of the variable in the superscript and its categories in the subscript) represent deviations from the overall mean η. There are three types of effects represented by τ parameters in Equation (1) (for more details see Hagenaars, 1993): (i) the main effect of the latent variable $\left(\tau_{w}^{W}\right)$ reflecting the ratio of the geometric mean of all cells sharing the same index w of the latent variable *W* divided by η, (ii) the main effect (e.g., $\tau_{a}^{A})$ of a specific category (e.g., *a*) of the manifest variables (e.g., *A*) reflecting the deviation of the geometric mean of all cells sharing index *a* from the overall geometric mean (η), and (iii) the interaction effects (e.g., $\tau_{aw}^{AW}$) of the latent variable (*W*) with one manifest variable (e.g., *A*) representing the ratio of the expected cell frequencies of the cell combination *a* and *w* to the product of the corresponding one-variable parameters. It is possible to inspect only the sub-table consisting of *A* and *W* (the cross-classification of *A* and *W*), because the collapsibility theorem holds in Equation (1) (Hagenaars, 1993). $\tau_{11}^{AW}$, for example, indicates the ratio to which the expected cell frequency of *a* = 1 and *w* = 1 is higher than predicted by the product of the overall geometric mean and the one-variable effects $\left(\eta \tau_{1}^{A}\tau_{1}^{W}\right)$. Using effect coding, the product of all parameters sharing the same superscripts is 1 $\left(\text{e}.\text{g}.,\;\prod_{w=1}^{W}\;\tau_{w}^{W}=\prod_{a=1}^{A}\;\tau_{a}^{A}=\prod_{w=1}^{W}\;\tau_{aw}^{AW}=\prod_{a=1}^{A}\;\tau_{aw}^{AW}=1\right)$. Additionally, in the standard latent class model local stochastic independence is assumed implying that there are no two or more-variable effects of observed variables. It is assumed that the two-variable effects between observed variables and the latent variable account for all associations between observed variables.

In standard applications, the number of latent classes is not a result of the model estimation but has to be specified prior to the analysis. If there is a strong theory postulating a specific number of latent categories (classes), this assumption could be tested in a confirmatory analysis. If, however, there is no theory about the number of latent categories, different models with different numbers of latent classes have to be estimated in an exploratory modeling approach and the goodness-of-fit coefficients have to be compared or a Dirichlet Process in a Bayesian estimation approach (Ferguson, 1973) can be used to estimate the number of latent categories. In all cases, the meaning of the latent classes depends on the model results (except for a priori restricted model parameters). Typical response patterns for the latent categories have to be identified, which may in turn be used to characterize individuals belonging to this latent class. This can best be done transforming the log-linear parameters into the (conditional response) probabilities of the latent class model (LCM; Habermann, 1979; Formann, 1992; Hagenaars, 1993; Heinen, 1993):

(2) $$\begin{array}{l} \pi_{w}^{W}=\frac{\tau_{w}^{W}}{\sum_{v=1}^{W}\tau_{v}^{W}},\\ \;(\text{with }v\text{ counting the latent categories of }W), \end{array}$$

and

(3) $$\begin{array}{l} \pi_{aw}^{AW}=\frac{\tau_{a}^{A}\tau_{aw}^{AW}}{\sum_{n=1}^{A}\;\tau_{n}^{A}\tau_{nw}^{AW}},\\ \;(\text{with }n\text{ counting the categories of }A). \end{array}$$

$\pi_{w}^{W}$ is the probability of latent class *w*, it depicts the proportion of individuals belonging to a particular class. $\pi_{aw}^{AW}$ is the conditional response probability indicating the probability that an individual belonging to class *w* scores in observed category *a*. The formalization as latent class probabilities [Equation (2)] provides the advantage that the parameters of the model can be more easily interpreted as the expected proportion of individuals belonging to a latent class. The conditional response probabilities [Equation (3)] allow for interpreting the meaning of the latent classes by considering the typical expected response pattern of individuals belonging to a particular class.

### Extension to a latent class model with two latent variables

The extension to models with more than one latent variable is straightforward (see e.g., Hagenaars, 1990, 1993) and can easily be done in the log-linear parameterization:

(4) $$\begin{array}{l} e_{abcdijklwy}=\eta \tau_{w}^{W}\tau_{y}^{Y}\tau_{wy}^{WY}\tau_{a}^{A}\tau_{b}^{B}\tau_{c}^{C}\tau_{d}^{D}\tau_{aw}^{AW}\tau_{bw}^{BW}\tau_{cw}^{CW}\tau_{dw}^{DW}\\ \;\tau_{i}^{I}\tau_{j}^{J}\tau_{k}^{K}\tau_{l}^{L}\tau_{iy}^{IY}\tau_{jy}^{JY}\tau_{ky}^{KY}\tau_{ly}^{LY}, \end{array}$$

where *W* and *Y* represent the two latent variables and *A* to *D* as well as *I* to *L* the two indicator sets of four manifest variables per latent variable. As can be seen from Equation (4), the two measurement parts are completely independent from each other implying that all associations between the observed variables can be explained by the associations between the latent variables and their influences on the manifest variables.

Modeling rater agreement at the latent level is straightforward adopting the rater agreement models for manifest variables to the latent level. In this case the latent variable *W* represents the true ratings of one group of raters and *Y* the true ratings of the other group of raters. However, three conditions have to be met: (i) the observed variables of the two methods have to measure the same construct (the indicators do not have to be identical but they should cover approximately the same construct), (ii) the number of latent categories principally has to be the same for the two methods, and (iii) the interpretation of the latent classes of the two latent variables must be identical or at least very close. For convenience, the ordering of the latent categories shall be the same for the two methods. Since the manifest variables do not interact with each other, we will only consider the model parameters at the latent level for ease of presentation.

*The latent saturated model* does not impose any restriction on the latent contingency table allowing for all cell specific latent interaction terms which indicate deviations of the expected frequencies from the expected cell frequencies based on the latent marginals only. This model is especially useful because it allows for all possible agreement and disagreement patterns and, moreover, represents an upper limit for the goodness-of-fit. The model equation is:

(5) $$e_{abcdijklwy}=\eta \tau_{w}^{W}\tau_{y}^{Y}\tau_{wy}^{WY}T_{\text{W}}T_{\text{Y}},$$

Where **T**_W_ and **T**_Y_ represent the two measurement parts of the model equation that include the one- and two-variable parameters with manifest variables. The inspection of the model parameters yields some insight into rater agreement. Examining the latent marginal distributions reveals if the two raters differ in the latent prevalence rates. Zwick (1988) states that the prevalence rates should not differ too much if the two variables represent the same construct. Agresti (1992) defines deviations in prevalence rates as an indication of rater bias. Additionally, the convergence (agreement) of the two methods can be determined by either inspecting the expected cell frequencies on the main diagonal or the log-linear two variable parameters for the latent variables affecting the cells on the main diagonal (where *w* = *y*). Finally, cells besides the main diagonal indicate disagreement. Comparing their expected frequencies to the expectation given the product of the overall geometric mean and the one-variable effects for the latent variable reveals if disagreement (in specific cell combinations) is more or less frequent than for independent ratings, the same information is given in the two-variable log-linear parameters for the latent variables.

The inspection of the expected cell frequencies and log-linear parameters in the latent saturated model is very informative but does not provide researchers with information if specific hypotheses about patterns of agreement or disagreement can be rejected. These hypotheses can be tested restricting the latent saturated model in meaningful ways yielding latent rater agreement models as the latent quasi-independence, the latent quasi-symmetry, and the latent symmetry models.

*The latent quasi-independence I and II models* are based on two assumptions: (i) There is no association between any pair of cells indicating disagreement between the two methods (raters) meaning that the independence model holds for these cells, and (ii) latent agreement cells are overrepresented with respect to the expectation given the lower order effects (the one-variable effects) meaning that the amount of agreement is higher than expected by chance alone. As a consequence, one might postulate that, if a quasi-independence model fits to the data, there are two types of answer processes at work (see also Schuster and Smith, 2006, discussing models for manifest variables). For one group of individuals the two latent variables are independent from each other. In this group, there is neither agreement nor disagreement above chance. Individuals of this group are therefore called ambiguous cases. The second group of individuals is characterized by perfect agreement and its individuals are called obvious cases. The quasi-independence I and II models are formally represented as:

(6) $$e_{abcdijklwy}=\eta \tau_{w}^{W}\tau_{y}^{Y}{\left(\zeta_{wy}^{WY}\right)}^{I}T_{W}T_{Y}\text{ with}\{\begin{array}{c} I=1\text{ if w}=y\\ I=0\text{ else} \end{array}$$

For cells besides the main diagonal the structural model simplifies to the product of the one-variable parameters $\left(\tau_{w}^{W}\tau_{y}^{Y}\text{ given }w\neq y\right)$, thus, the independence assumption accounts for disagreement cells. For cells on the main diagonal an additional parameter $\left(\zeta_{wy}^{WY}\right)$ is introduced to represent the amount of true agreement above chance. In the quasi-independence I model, the cell frequencies on the main diagonal may differ from cell to cell. Differences in $\left(\zeta_{wy}^{WY}\right)$ reflect that for some categories there are relatively more obvious cases than for other categories. In the quasi-independence II model, $\left(\zeta_{wy}^{WY}\right)$ is fixed to be constant across all cells, therefore the ratio of true agreement and agreement on chance is the same for all categories, and, hence, the ratio of obvious to ambiguous cases is identical across categories. With respect to the analysis of convergent validity these models imply, that the two methods partially converge since there is an overrepresentation for cells on the main diagonal. Additionally, it is assumed that the two methods (raters) do not confound any categories systematically since their scores are independent from each other for disagreement cells.

*The latent quasi-symmetry and latent symmetry models* do not imply the strong assumption of independent ratings besides the main diagonal as do the quasi-independence models, but allow that raters can systematically confound categories. This confusion is supposed to be symmetric. For example, the combination of ratings *w* = 1 and *y* = 2 is exactly equally more frequently expected as the combination *w* = 2 and *y* = 1 compared to the expectations given the lower order effects. The model allows for latent two-variable effects for cells on the main diagonal but also for over- and underrepresentation of disagreement cells. The quasi-symmetry model is defined by:

(7) $$e_{abcdijklwy}=\eta T_{w}T_{y}\tau_{w}^{W}\tau_{y}^{Y}\tau_{wy}^{WY},\text{ with }\tau_{wy}^{WY}=\tau_{yw}^{WY},$$

The quasi-symmetry and symmetry models do not only address higher agreement rates but also provide some information with respect to the interchangeability of raters. If the model fits to the data, raters confound latent categories in a symmetrical way, that is, they are interchangeable with respect to their confounding of categories.

If additionally, the latent one-variable effects do not differ between raters the more restrictive assumptions of the symmetry-model hold. In contrast to the quasi-symmetry model, the marginal distributions must not differ between raters leading to the same prevalence rates and to a complete interchangeability of raters (Agresti, 1992).

### Implications of the different rater agreement models

Since the interpretation of the log-linear parameters is not the same in the different rater agreement models, we will consider proportions in the remainder of the text. Proportions can be determined by the ratio of a specific expected frequency and the total sample size.

Latent rater agreement models allow for assigning individuals rated by the two (or more) raters to latent classes. Given the probabilistic nature of the model this assignment is affected with a specific error. An individual is assigned to the latent class for which her or his assignment probability is highest. The mean assignment probability of all individuals who are assigned to a particular class, that is the average of the assignment probabilities, yields the reliability of the class assignments.

The latent marginal proportions can be inspected in order to examine differences in the latent prevalence rates. Large differences in these proportions reveal that the two methods are biased with respect to each other. The *Method Bias I (MB*1) coefficient shows to which ratio the two raters differ with respect to their latent distributions:

(8) $$MB1_{\left(wy\right)}=\frac{\pi_{w}^{W}}{\pi_{y}^{Y}},\text{ for }w=y.$$

This definition of method bias is similar to the conception of method bias in standard log-linear models for rater agreement (Agresti, 1992). Note, that this bias is not defined as a bias indicating differences/deviations from the true status or the true distribution of the latent variable but as a bias with respect to the other rater. Values larger than 1 indicate that the rater whose latent variable is in the numerator uses this category more frequently than the other rater. Values below 1 indicate the opposite. High (or low) values on *MB*1 indicate that the two raters do not perfectly agree on the prevalence rates and therefore also indicate a cause of a lack of convergent validity. One may test for the latent method bias by comparing models with equality restrictions on the latent marginal proportions (for all categories or only for some categories) to models without these restrictions.

The different latent rater agreement models have different implications with respect to the structure of *agreement* and *disagreement*. The least restrictive model is the saturated model allowing for all cell-specific associations in the latent bivariate table. The quasi-independence models are rather restrictive models presuming that the two latent variables are associated with respect to agreement cells but independent from each other for disagreement cells. The symmetry models imply a symmetric structure of disagreement and, thus, allow for higher or lower disagreement rates.

The convergent validity of two methods (raters) can be defined as agreement in rater agreement models. Thus, high expected cell proportions on the main diagonal compared to the product of the latent marginal cell proportions indicate higher *agreement rates* or *convergence*. Note, that the agreement rates (CO; convergence) may differ between categories:

(9) $$CO_{\left(w\right)}=\frac{\pi_{wy}^{WY}}{\pi_{w}^{W}\pi_{y}^{Y}}\text{ for }w=y.$$

A value larger than 1 indicates higher agreement than expected based on the marginals. Comparing the convergence indices across categories allows for an inspection if there is general agreement or if there is rather a category specific agreement, moreover, the assumption of constant (i.e., category unspecific) agreement can be tested fitting a constant two-variable parameter for cells on the main diagonal as in the Quasi-Independence II model, for example.

The distinguishability index:

(10) $$Dist_{\left(wy\right)}=\frac{\pi_{wy}^{WY}}{\pi_{w}^{W}\pi_{y}^{Y}},\text{ for }w\neq y,$$

depicts the ratio to which proportions of specific cell combinations besides the main diagonal deviate from the expected proportions given the one-variable effects. Values smaller than 1 indicate that raters can well distinguish between the corresponding categories. Values larger than 1 indicate that raters confound these two categories. The distinguishability index is only meaningful in the quasi-symmetry and symmetry as well as in the saturated model, because, in the quasi-independence models, the expected proportions besides the main diagonal only depend on the marginal distributions of ambiguous cases. The distinguishability index becomes meaningful in the former models when cells besides the main diagonal are modeled. In the saturated model, the distinguishability index can statistically be tested inspecting the associated *p*-value of the corresponding log-linear effect.

## The multitrait-multimethod latent class model

The latent rater agreement models described so far can be used to determine the convergence of different ratings and the distinguishability of the different categories representing one particular construct. However, convergence and distinguishability may be a general property of the two methods or specific to the considered construct. Therefore, it is important to include additional constructs into the analysis. Analyzing more than one construct measured by more than one method in a multimethod latent class model corresponds to the multitrait-multimethod (MTMM) analysis as proposed by Campbell and Fiske (1959).

In addition to the examination of convergence and distinguishability, MTMM latent class models (LCM) allow for an analysis of discriminant validity. Distinguishability and discriminant validity are two related yet different concepts. Distinguishability is the degree to which non-corresponding categories can be separated by two or more raters, discriminant validity is the degree to which two constructs are unrelated. Discriminant validity is high, if there are low associations between the categories of the two constructs.

The MTMM LCM corresponds to a combination of four log-linear measurement models with latent variables:

(11) $$\begin{array}{l} e_{WXYZwxyz}\;=\;\eta T_{W}T_{X}T_{Y}T_{Z}\tau_{w}^{W}\tau_{x}^{X}\tau_{y}^{Y}\tau_{z}^{Z}\\ \;\times \tau_{wx}^{WX}\tau_{wy}^{WY}\tau_{wz}^{WZ}\tau_{xy}^{XY}\tau_{xz}^{XZ}\tau_{yz}^{YZ},\\ \;\times \tau_{wxy}^{WXY}\tau_{wxz}^{WXZ}\tau_{wyz}^{WYZ}\tau_{xyz}^{XYZ}\tau_{wxyz}^{WXYZ} \end{array}$$

where **T**_**W**_ and **T**_**Y**_ represent two measurement models of two raters rating the same construct and **T**_**X**_ and **T**_**Z**_ represent the two measurement models of the same two raters rating the other construct. The measurement models are the same models as described above. *W* and *Y* represent the latent variables of the first construct (say neuroticism) measured by two methods (*W*, self-reported neuroticism; *Y*, peer reported neuroticism); the latter two latent variables may represent self-reported conscientiousness (*X*) and peer reported conscientiousness (*Z*). Again, all indicators are locally stochastically independent from each other. At the level of the latent variables, two-, three-, and four-variable interactions are possible. Therefore, the analysis of convergent and discriminant validity is not restricted to the analysis of bivariate relationships but can be extended to higher order effects. In the remainder, we will discuss the most meaningful interactions with respect to convergent and discriminant validity. A detailed descriptions of all effects can be found in Nussbeck (2009). Additionally, we will refer to the saturated model at the level of latent variables in order to present all possible effects. However, meaningful restrictions as for the latent rater agreement models can be easily incorporated.

The cells of the latent frequency table fall into three parts: (a) Cells indicating *complete agreement*, that is agreement on both constructs (e.g., *w* = *y* and *x* = *z*), (b) cells indicating *partial agreement*, that is agreement on one construct (e.g., *w* = *y* and x ≠ *z*), and (c) cells indicating disagreement on both constructs (*w* ≠ *y* and x ≠ *z*). All cell proportions are influenced by the complete set of one-, two-, three-, and four-variable effects in the saturated model. We will discuss the meaning of the different interaction effects with respect to the order of the effects:

### Four-variable effects

In the latent saturated model, the four variable effects can be classified as representing three different properties. The four variable effect can represent rater agreement and disagreement, but can also reflect the discriminant validity of the different constructs.

### Complete agreement

The four-variable log-linear parameters representing agreement on *both* constructs indicate the judgeability of the targets (Funder, 1995). If all four-variable parameters for complete agreement cells are larger than 1 and of equal size, this indicates that the convergence of the two raters is stable across the different category combinations. The odds to agree given the expected proportions based on lower order effects are identical on all category combinations indicating agreement on both constructs. This overall agreement rate may be due to two reasons (see Funder, 1995): There is a group of individuals who are easily judgeable (good targets), or the two constructs are especially visible in some targets (*palpability*), the concept of palpability differs from the concept of good targets to the extent that palpability refers to the construct and, hence, the same individuals are less well judged on other constructs whereas good targets principally produce high agreement rates irrespective of the construct. If the agreement rate is constant the judgeability of the targets or the palpability does not depend on the scores on one of the latent constructs (it is constant across all categories).

As a second case of complete agreement, all four-variable parameters for complete agreement cells may be larger than 1 but differ from each other. In this case, the raters agree more often than expected based on the lower order effects. Judgeability of targets depends partly on their status on the latent variables. Individuals who belong to a special easily judgeable category of one trait can be more easily accurately (congruently) judged on a category of the other traits as well. In this case, judgeability (as palpability) is a property of different constellations of the latent categories. Being extraverted may be part of properties characterizing good targets by rendering other constructs more accessible to observations.

In the third case, there are only few but very large four-variable parameters for complete agreement cells low discriminant validity is found. The latent categories of the different constructs partly overlap and cannot be considered very distinct from each other.

### Partial agreement

Four-variable parameters of cells indicating agreement on one construct but not on the other represent a special kind of *rater bias*. If the four-variable parameters of cells indicating agreement on one but not on the other construct are larger than 1, raters agree on one construct but they disagree systematically on the other construct. This may be the case for raters who agree on a target person's extraversion but who have different views or theories about the relation between extraversion and intelligence, for example. One rater may assume that moderately extraverted individuals also tend to be more intelligent while the other assumes moderately extraverted individuals to be very intelligent.

The four-variable parameters of (particular or all) cells indicating agreement on one but not on the other construct are smaller than 1. In this case, disagreement between the two raters with respect to specific category combinations is underrepresented if they agree on the other construct. This pattern may be expected as a byproduct of high overall agreement rates. This effect thus shows (if there is agreement) that there is higher agreement on one construct (on all or on one category) if there is agreement on the other one.

### Disagreement

The latent four-variable parameters of cells besides the agreement and partial agreement cells represent influences which may be due to bias or to general disagreement. Some four-variable parameters for complete disagreement cells are larger than 1. In this case, particular combinations of one rater's latent scores are associated to the other rater's scores but for different cell combinations. If raters weigh some behavioral cues in different ways given cues on the other construct they may be more often categorized in latent disagreement cells. If, for example, one rater classifies an individual due to specific behavioral cues as highly extraverted and, additionally, these cues may lead this rater to also classify this individual as moderately neurotic this combination of behavioral cues may be associated to the moderately extraverted and highly neurotic classes for the other rater.

Some four-variable parameters for complete disagreement cells are smaller than 1. This may in most cases be due to higher complete and/or partial agreement rates. Therefore, higher agreement also affects the disagreement cells in the saturated model. Yet, this may also be due to high disagreement rates in a different cell combination.

### Three-variable effects

As other lower order effects, three-variable effects may be interpreted as average effects influencing particular cell combinations. The interpretation of these effects is only meaningful if the higher order effects are absent or have the same qualitative impact (increase or decline of the expected frequencies) on the cells affected by the lower order effect. The same qualitative impact implies that all higher order effects lead to a higher co-occurrence of the category combinations of the lower order effects and the lower order effects may be interpreted as average effects. For sake of simplicity, consider the case of absent higher order effects.

Log-linear parameters do not impose any directional link. The effects presented here correspond to correlations and higher order correlations; therefore, it is principally possible to interpret all effects as the influences of any variable on the association of the other two variables. In order to examine rater agreement as a special form of convergent validity it is useful to inspect the meaning of the latent three-variable effects as the influence of one latent construct's score on the joint categorization of the other construct. Therefore, these effects can be interpreted in two principal ways. Three-variable effects either represent properties of judgeable individuals or sources (correlates) of disagreement. These influences are especially meaningful in models when one rater can be conceived as providing better ratings than the other but may also occur in other cases.

### Agreement

The three-variable parameters of cells representing agreement on one construct depict if agreement depends on the category of the other construct. If the three-variable parameters of cells representing agreement on one construct are high for specific categories of one variable of the other construct, then the three-variable effects indicate for which specific categories of *X* agreement on *W* and *Y* is obtained to a higher degree than expected based on the lower order effects. The categories of *X* can be conceived as a kind of judgeability indicator or as marker categories for good targets. This interpretation is especially meaningful if one rater (providing the *X* score) can be conceived as a better rater of the individual's true status than the other.

### Disagreement

The three-variable parameters of cells representing disagreement on one construct depict if this disagreement is associated to the status on the other construct. If the three-variable parameters of cells representing disagreement on one construct and a particular category of the other construct are large than one, then the expected frequencies are higher for a specific case of disagreement if a particular category is chosen on the other construct. This constellation (e.g., the expected proportion is high for “not extraverted” and “not neurotic” in self-ratings and “neurotic” in peer ratings) represents rater bias of the peer with respect to the self-rating confounding neurotic with not neurotic but only for extraverted.

### Two- and one-variable effects

Two- and one-variable effects are especially meaningful with respect to agreement rates, distinguishability of categories, and method bias. If there are no higher order effects, the one-variable effects can directly be interpreted with respect to *MB*1 and the two-variable parameters can be directly interpreted in a similar way as the criteria introduced by Campbell and Fiske (1959).

Agreement and disagreement can be inspected based on the log-linear parameters as described for the latent rater agreement models. Additionally, the distinguishability index reveals which categories are more frequently confounded and which categories can be neatly distinguished.

The inspection of two variable effects for categories of different constructs measured with the same method allows also for an analysis of heterotrait-monomethod associations (sensu Campbell and Fiske, 1959). In general, this effect should be rather weak (close to 1) to indicate discriminant validity for all of these two-variable effects. However, special categories of neuroticism (highly neurotic) may, for example, co-occur with particular categories of conscientiousness (moderately conscientious) but not with others and therefore show a substantial two-variable effect. This effect may be due to several (interacting) influences: a theoretical overlap of the categories (a theoretically meaningful category combination; yet, the constructs are not perfectly discriminant), and/or method bias. Method bias is a rater specific view of how categories belonging to two different constructs are related. These effects do not have to be identical across the different raters.

The associations between variables belonging to different constructs judged by different raters correspond to *heterotrait-heteromethod* associations sensu Campbell and Fiske (1959). The corresponding latent two-variable parameters mirror associations between the latent constructs that are shared between raters. These effects can be due to a theoretical overlap of the constructs but they cannot be due to method bias. Therefore, the ratio of the association between traits belonging to one rater (confounded with bias) and the mean association of the corresponding bias free associations indicates the rater specific bias type 2 (*MB*2; the rater's view that is, not shared across raters):

(12) $$MB2_{\left(WX\right)}=\frac{\pi_{wx}^{WX}}{\sqrt{\pi_{wz}^{WZ}\pi_{xy}^{XY}}}.$$

The denominator gives the expectancy of the bias free association taking the geometric mean of the associations across the two raters. The association between the same categories within one method containing influences due to “true” associations but also due to bias is compared to this “average bias free association.” The method bias type II depends on three proportions: The heterotrait-monomethod cell proportion and the two heterotrait-heteromethod cell proportions representing the same latent categories. Since the denominator is the geometric mean of the two heterotrait-heteromethod proportions this index should not be calculated if the heterotrait-monomethod proportion falls into the interval between the two heterotrait-heteromethod proportions. In this case, the rater-specific view is in the “middle” of the rater-unspecific views, it is, hence, not higher or lower as the error free interaction and is, therefore, not biased. Values larger than 1 indicate an association of the two categories for one rater that goes beyond the bias-free association. That is, one rater implicitly or explicitly associates the two categories to a greater extent than do different raters. It reflects rater specific theories or beliefs about the combined prevalences of different statuses (e.g., halo-effect). Values smaller than 1 indicate that this association is less frequently expected than based on the bias-free association—which may be interpreted as an inversed halo-effect. This coefficient is theoretically founded in the postulate of Campbell and Fiske (1959) that the pattern of associations should be the same for all traits in monomethod as well as in heteromethod blocks.

*The interpretation of all parameters but the highest-order parameters* as presented here can only be done if all higher order effects are absent. However, dealing with empirical data researchers are interested in the agreement rates of their raters. The latent log-linear parameters of lower order effects correspond to “average” effects. Therefore, these effects should only be interpreted (as a directional effect not interpreting the parameter value) if the higher order interactions do not change the direction of the main (lower order) effect for different categories (all parameters of the considered cells must be larger or smaller than 1).

A heuristic inspection of latent bivariate subtables can be done to get some insight into convergent and discriminant validity sensu Campbell and Fiske (1959). However, if higher order effects are present, the tables are not collapsible. Therefore, we do not recommend inspecting the log-linear parameters of bivariate subtables in cases where higher order effects are present. However, probabilities and conditional probabilities may be calculated and compared to get an estimation of convergent and discriminant validity as well as agreement and disagreement rates. Restricting the underlying log-linear model allows to reveal the latent underlying structure.

## Empirical application

In this section, the multimethod latent class models will be illustrated by an empirical application. In this application, 480 self-reports and peer reports with respect to four items measuring neuroticism (*NS*, neuroticism self-report; *NA*, neuroticism peer report; items are: vulnerable, sensitive, moody, and self-doubtful) and conscientiousness (*CS*, conscientiousness self-report; *CA*, conscientiousness peer report; items are: industrious, diligent, dutiful, and ambitious) on a three-categorical agreement scale (not at all, fairly, very much so) will be analyzed. We assumed that—in accordance with the number of categories of an item—the number of latent classes per latent variable equals three. We estimated models with two-, three-, and four-variable effects as highest order effects using the computer program LEM (Vermunt, 1997a).

Since the χ^2^ approximations are not trustworthy due to sparse tables, the goodness of fit of the models can be compared relying on information criteria as AIC and BIC. The model with two-variable interactions as highest order interactions (an annotated LEM-input can be found in the Supplementary Material) fit best to the data (AIC = −86086704; BIC = −265574236 vs. AIC = −86086694; BIC = −265574093 for the solution with three-variable interactions and AIC = −86086669; BIC = −265574001 for the solution with four-variable interactions).

Since there are too many log-linear parameters on the boundaries of the parameter space we will present the conditional response probabilities which can still be interpreted (e.g., Galindo-Garre and Vermunt, 2005). Tables 1, 2 depict the conditional response probabilities for the four measurement models. The three latent classes for the two latent variables for neuroticism (self- and peer report) can roughly be characterized as not neurotic, moderately neurotic, and highly neurotic classes. For the self-ratings, the first (low neuroticism) class consists of 25% of the sample, the second (moderate neuroticism) class of 42%, and the third (high neuroticism) class consists of 33%. The peer ratings do not differ with respect to the proportions of classifications (25, 41, and 34%, respectively). The three latent classes for conscientiousness can be classified as not being conscientious, being moderately conscientious, and being highly conscientious. The class proportions are 24, 35, and 41% for the self-ratings as well as 17, 31, and 52% for the peer ratings.

**Table 1:** **Conditional response probabilities of the manifest response categories for the construct neuroticism**.

**Variable**	**Manifest categories**	**Latent class**					
		***ns* = 1**	***ns* = 2**	***ns* = 3**	***na* = 1**	***na* = 2**	***na* = 3**
	1	0.30	0.03	0.01	0.56	0.01	0.04
Vulnerable	2	0.44	0.08	0.04	0.34	0.52	0.00^*^
	3	0.26	0.89	0.95	0.10	0.47	0.96
	1	0.48	0.03	0.00^*^	0.70	0.15	0.00^*^
Sensitive	2	0.44	0.11	0.02	0.30	0.50	0.10
	3	0.08	0.86	0.98	0.00^*^	0.35	0.90
	1	0.66	0.51	0.00	0.69	0.63	0.32
Moody	2	0.20	0.50	0.04	0.21	0.29	0.27
	3	0.14	0.00	0.96	0.10	0.08	0.40
	1	0.52	0.22	0.10	0.68	0.48	0.25
Doubtful	2	0.30	0.16	0.13	0.20	0.30	0.27
	3	0.18	0.62	0.78	0.12	0.22	0.48

* *Boundary values. ns, categories of the latent variable for neuroticism (self-report); na, categories of the latent variable for neuroticism (peer report)*.

**Table 2:** **Conditional response probabilities of the manifest response categories for the construct conscientiousness**.

**Variable**	**Manifest categories**	**Latent class**					
		***cs* = 1**	***cs* = 2**	***cs* = 3**	***ca* = 1**	***ca* = 2**	***ca* = 3**
	1	0.76	0.02	0.01	0.84	0.07	0.00
Industrious	2	0.19	0.79	0.05	0.15	0.72	0.03
	3	0.05	0.19	0.93	0.02	0.21	0.97
	1	0.88	0.09	0.00^*^	0.91	0.03	0.01
Diligent	2	0.11	0.81	0.05	0.07	0.82	0.02
	3	0.02	0.09	0.95	0.03	0.15	0.97
	1	0.21	0.02	0.01	0.47	0.09	0.00
Dutiful	2	0.33	0.27	0.05	0.33	0.42	0.07
	3	0.46	0.71	0.94	0.20	0.49	0.92
	1	0.69	0.20	0.05	0.86	0.26	0.01
Ambitious	2	0.26	0.59	0.11	0.13	0.60	0.16
	3	0.05	0.21	0.84	0.01	0.14	0.83

* *Boundary values. cs, categories of the latent variable for conscientiousness (self-report); ca, categories of the latent variable for conscientiousness (peer report)*.

Table 3 presents the expected proportions of the latent joint distribution. Expected cell proportions that differ for at least 2% from the product of the latent marginals are depicted in bold type. In total, the entries of 19 cells (out of 81 cells) are bold typed. Seven out of these 19 represent overall agreement (dark gray cells). Eight cells represent partial agreement (light gray or surrounded subtables) and 4 represent total disagreement. Overall agreement cells comprise about 27% of the sample, the highest entries can be found for the agreement combinations of highly conscientious with moderately or highly neurotic (14% of all entries fall into these two joint categories). The agreement rates are principally higher for individuals who are at least moderately conscientious and at least moderately neurotic. Partial agreement for conscientiousness (26%) can primarily be found for highly conscientious individuals (16% of the joint judgments). Similarly, partial agreement for neuroticism (20%) can primarily be found for moderately or highly neurotic individuals (15% of the joint judgments).

**Table 3:** **Cross-classification of the latent categories for neuroticism and conscientiousness in the CT MTMR Model with two-variable effects as highest order interactions**.

			***CA***		
			**1**	**2**	**3**
		*CS* = 1	**0.02 (0.00)**	**0.02 (0.00)**	0.00 (0.00)
	*NA* = 1	*CS* = 2	0.01 (0.00)	**0.02 (0.00)**	0.01 (0.00)
		*CS* = 3	0.00 (0.00)	0.01 (0.00)	**0.02 (0.00)**
		*CS* = 1	0.00 (0.00)	0.01 (0.00)	0.01 (0.00)
*NS* = 1	*NA* = 2	*CS* = 2	0.00 (0.00)	0.01 (0.00)	**0.02 (0.00)**
		*CS* = 3	0.00 (0.00)	0.00 (0.00)	**0.04 (0.00)**
		*CS* = 1	0.00 (0.00)	0.00 (0.00)	0.00 (0.00)
	*NA* = 3	*CS* = 2	0.00 (0.00)	0.01 (0.00)	0.01 (0.00)
		*CS* = 3	0.00 (0.00)	0.00 (0.00)	0.01 (0.00)
		*CS* = 1	0.01 (0.00)	0.01 (0.00)	0.00 (0.00)
	*NA* = 1	*CS* = 2	0.01 (0.00)	0.01 (0.00)	0.00 (0.00)
		*CS* = 3	0.00 (0.00)	0.01 (0.00)	0.01 (0.00)
		*CS* = 1	0.01 (0.00)	0.01 (0.00)	0.01 (0.00)
*NS* = 2	*NA* = 2	*CS* = 2	0.01 (0.00)	**0.02 (0.00)**	**0.04 (0.01)**
		*CS* = 3	0.00 (0.00)	0.01 (0.00)	**0.09 (0.01)**
		*CS* = 1	0.01 (0.00)	0.01 (0.00)	0.01 (0.00)
	*NA* = 3	*CS* = 2	0.01 (0.00)	**0.02 (0.00)**	0.02 (0.00)
		*CS* = 3	0.00 (0.00)	0.01 (0.00)	**0.05 (0.01)**
		*CS* = 1	0.01 (0.00)	0.02 (0.00)	0.00 (0.00)
	*NA* = 1	*CS* = 2	0.01 (0.00)	0.01 (0.00)	0.00 (0.00)
		*CS* = 3	0.00 (0.00)	0.01 (0.00)	0.01 (0.00)
		*CS* = 1	0.00 (0.00)	0.01 (0.00)	0.01 (0.00)
*NS* = 3	*NA* = 2	*CS* = 2	0.00 (0.00)	0.01 (0.00)	**0.02 (0.00)**
		*CS* = 3	0.00 (0.00)	0.01 (0.00)	**0.04 (0.00)**
		*CS* = 1	0.01 (0.00)	0.02 (0.00)	0.01 (0.00)
	*NA* = 3	*CS* = 2	0.01 (0.00)	**0.03 (0.00)**	**0.02 (0.00)**
		*CS* = 3	0.00 (0.00)	0.01 (0.00)	**0.05 (0.00)**

*Entries in bold type depict expected proportions that deviate from the predictions based on the marginals by more than one decimal. Entries in parentheses represent the product of the latent marginals. NS, neuroticism (self-report); NA, neuroticism (peer report); CS, conscientiousness (self-report); CA, conscientiousness (peer report). Dark gray cells represent overall agreement; light gray cells indicate partial agreement for conscientiousness, surrounded subtables indicate partial agreement for neuroticism*.

A more thorough insight into the interplay of the four latent variables can be gained by inspecting the bivariate latent distributions (see Table 4). Since we specified no three- or four-way interactions, we can inspect the two-way frequency tables (the bivariate joint distributions are exactly reproduced). We will exemplarily present the cross-tabulations of monotrait-heteromethod tables, heterotrait-monomethod tables, and heterotrait-heteromethod tables in order to exemplify all possible constellations. Table 4 presents the latent rater agreement sub-model for neuroticism in the upper left corner. In order to compare the two model-implied latent marginal distributions for neuroticism with each other, the method bias type I can be determined. The coefficients [*MB*1_(ns = 1, na = 1)_ = 1.00; *MB*1_(ns = 2, na = 2)_ = 1.03; *MB*1_(ns = 3, na = 3)_ = 0.98] show that the two raters yield ratings with almost perfectly the same prevalence rates. Inspecting the cells on the main diagonal shows considerable agreement. The category-specific agreement rates are in the range of 1.27–1.92 (see Table 5) with the highest value for the latent cell combination of not being neurotic. These effects are comparable to the monotrait-heteromethod effects sensu Campbell and Fiske (1959).

**Table 4:** **Bivariate cross-classification of the latent variables: estimated relative frequencies**.

		***NA***				***CS***				***CA***			
		**1**	**2**	**3**		**1**	**2**	**3**		**1**	**2**	**3**	
	1	0.12 (0.06)	0.09 (0.10)	0.04 (0.09)	0.25	0.08 (0.07)	0.09 (0.09)	0.09 (0.11)	0.26	0.05 (0.04)	0.07 (0.08)	0.12 (0.13)	0.25
*NS*	2	0.06 (0.11)	0.22 (0.17)	0.14 (0.14)	0.42	0.08 (0.11)	0.16 (0.15)	0.18 (0.17)	0.42	0.07 (0.07)	0.12 (0.13)	0.24 (0.21)	0.42
	3	0.07 (0.08)	0.10 (0.14)	0.16 (11)	0.33	0.10 (0.08)	0.10 (0.12)	0.13 (0.14)	0.33	0.05 (0.06)	0.12 (0.11)	0.16 (0.17)	0.33
		0.25	0.41	0.34		0.25	0.35	0.41		0.17	0.31	0.52	
	1					0.09 (0.06)	0.09 (0.09)	0.07 (0.10)	0.25	0.08 (0.04)	0.10 (0.08)	0.07 (0.13)	0.25
*NA*	2					0.08 (0.10)	0.14 (0.14)	0.20 (0.17)	0.41	0.03 (0.07)	0.10 (0.13)	0.28 (0.21)	0.41
	3					0.07 (0.09)	0.13 (0.12)	0.19 (0.14)	0.34	0.05 (0.06)	0.12 (0.11)	0.18 (0.18)	0.34
						0.24	0.35	0.41		0.17	0.31	0.52	
	1									0.08 (0.04)	0.10 (0.07)	0.06 (0.12)	0.24
*CS*	2									0.07 (0.06)	0.14 (0.11)	0.15 (0.18)	0.35
	3									0.02 (0.07)	0.07 (0.13)	0.32 (0.21)	0.41
										0.17	0.31	0.52	

*NS, neuroticism (self-report); NA, neuroticism (peer report); CS, conscientiousness (self-report); CA, conscientiousness (peer report); values in parentheses represent the product of the latent marginals*.

**Table 5:** **Distinguishability indices and category specific agreement rates**.

		***NA***		
		**1**	**2**	**3**
	1	1.92	0.88	0.47
*NS*	2	0.57	1.27	0.99
	3	0.86	0.75	1.40
		***CA***		
		**1**	**2**	**3**
	1	2.03	1.37	0.44
*CS*	2	1.12	1.25	0.81
	3	0.30	0.56	1.49

*NS, neuroticism (self-report); NA, neuroticism (peer report); CS, conscientiousness (self-report); CA, conscientiousness (peer report); distinguishability indices can be found besides the main diagonal; category specific agreement rates can be found upon the main diagonal*.

The inspection of the disagreement cells besides the main diagonal also shows an interesting pattern. All but one cell (*ns* = 2 and *na* = 3) show lower expected proportions than would be expected based on the latent marginals. The distinguishability indices in Table 5 reflect that the cell combinations (*ns* = 2 and *na* = 1) and (*ns* = 1 and *na* = 3) are only about half as often expected than predicted by the product of the marginals. For example, self-rated not neurotic individuals are rarely judged to be neurotic by the peer rater (*Dist*_(1.3)_ = 0.47). In the same vein, moderately neurotic individuals are less often rated not neurotic (*Dist*_(2.1)_ = 0.57). Peers obviously perceive if individuals show neurotic behavior tendencies (self-rated). They also do not overestimate the self-rated neuroticism score producing no overestimation for the combination of moderately neurotic in the self-report and neurotic in the peer report (*Dist*_(2.3)_ = 0.99), however, peers also do not distinguish between these latter categories. All other distinguishability indices show that self- and peer raters show lower disagreement, yet, they do not differ vastly from the product of the latent marginals (absolutely and relatively). Self-raters and peers discriminate fairly well between the different categories of neuroticism.

Table 4 also presents the latent cross classification of the two latent variables representing conscientiousness (lower right subtable). The method bias type I coefficients [*MB*1_(*cs* = 1, *ca* = 1)_ = 1.41; *MB*1_(*cs* = 2, *ca* = 2)_ = 1.13; *MB*1_(*cs* = 3, *ca* = 3)_ = 0.79] reveal that self- and peer raters deviate considerably in their latent marginals. Peers rate the targets in more than half of the times as highly conscientious (1.27 times more often than the self-raters). Self-raters choose the lower categories more often. This finding may be due to the fact that the self-raters are almost exclusively students. In order to successfully complete one's studies a specific level of conscientiousness is required, peers may attribute the fact that self-raters complete their work as students to their personality whereas the self-raters may compare themselves to others and do not perceive themselves as conscientious. Moreover, they know about their own possible difficulties in completing the work (e.g., procrastination) and therefore rate themselves lower on conscientiousness. One might conclude that better (more diverse) information is needed for the peer raters to achieve higher agreement rates. The entries on the main diagonal also show agreement of the two raters with respect to conscientiousness (high convergent validity).

The latent joint classification of the latent variables of the self-reports (self-reported neuroticism and conscientiousness, upper subtable in the mid-column) show little deviations from the expected proportions and the product of the latent marginals. This indicates that the self-raters distinguish well between the two latent constructs. For self-raters, these constructs are not associated. This indicates almost perfect discriminant validity sensu Campbell and Fiske (1959).

Peers on the other hand perceive the two constructs as related rating other individuals (see Table 4, lower table in mid-column). With respect to the peer ratings the combination of not being neurotic and highly conscientious appears less often than predicted based on the marginals. Peers rather tend to choose the first categories on both variables. Additionally, they perceive moderately neurotic individuals as highly conscientious and less frequently as not conscientious or moderately conscientious. Therefore, one may conclude that there is a lack of discriminant validity with respect to these two traits for peer ratings. However, this lack only concerns particular categories and does not generalize across all possible constellations because the other combinations do not deviate to a great extent from the product of their marginals.

The latent cross-classification of the self-rated neuroticism-scores and the peer rated conscientiousness-scores again shows no deviation from the product of the latent marginals (see Table 4; subtable in the middle of the last column). Self- and peer ratings of the different constructs are completely distinct from each other. This indicates high discriminant validity across raters. However, this is not true for the latent cross-classification of the peer rated neuroticism and the self-rated conscientiousness. The two latent trait variables (*NA* and *CS*) are associated to a stronger degree than the opposite combination. If self-raters perceive themselves as highly conscientious peers do no longer tend to judge them not neurotic but choose the middle and high category of neuroticism. That is, high conscientiousness in self-ratings is slightly confounded with neuroticism in the peer view.

Table 6 presents the method bias type II parameters for the self-report. Empty cells indicate parameters that are meaningless since the monomethod association is in the range of the two heteromethod associations. Self-raters tend to rate themselves as highly neurotic but not conscientious, moderately neurotic but not conscientious, and not neurotic and moderately conscientious more often than on average. The self-raters conceive themselves less frequently as highly neurotic combined with highly conscientious or moderately conscientious than predicted by the average ratings. The same is true for moderately neurotic individuals who perceive themselves not as often as highly conscientious as predicted by the joint ratings.

**Table 6:** **Method bias type II coefficients**.

			***CS***	
		**1**	**2**	**3**
	1	–	1.07	–
*NS*	2	1.21	1.26	0.85
	3	1.62	0.84	0.75
			***CA***	
		**1**	**2**	**3**
	1	–	1.26	0.70
*NA*	2	0.48	0.75	1.29
	3	0.80	0.94	–

*NS, neuroticism (self-report); NA, neuroticism (peer report); CS, conscientiousness (self-report); CA, conscientiousness (peer report)—indicates that MB2 is meaningless in this cell*.

A completely different picture is given for the bias of peer ratings. The combinations of not neurotic and not conscientious as well as highly neurotic and highly conscientious are not biased with respect to the joint ratings. A positive bias (overrepresentation) can be found for the combinations of not neurotic and moderately conscientious as well as moderately neurotic and highly conscientious. All other cells are less frequently expected than predicted by the joint ratings. Peers do not associate low conscientiousness to the latent classes being sensitive but stable (i.e., moderately neurotic) or neurotic as expected by the average association. The same is true for the combination of not neurotic and highly conscientious.

Self-raters and peers thus differ with respect to the cells that are over- or underrepresented in the cross-classification of their latent variables. Peers perceive the targets principally as more conscientious (see method bias type I) than do self-raters. Additionally, they show larger expected frequencies for two particular cell-combinations of the latent traits. That is, moderately neurotic self-rated individuals are rated more often as highly conscientious by peers compared to the self-ratings and not neurotic individuals are rated more often as moderately conscientious. These combinations are not overrepresented in the self-report. Therefore, these coefficients reflect a view that is specific to the peer raters. In the same vein, the peers show under-representations of the cells for ratings combining not conscientious with moderately or highly neurotic. Again, this underrepresentation is specific to the view of peers because self-raters show overrepresented ratings for these categories. The two raters also differ with respect of their views concerning the association of targets being moderately conscientious and moderately neurotic. While self-raters choose this category combination more often than could be expected relying on the bias-free associations (between raters) peers tend to underestimate this association.

## General discussion

The aim of the current contribution is to present latent rater agreement models as well as MTMM models for the analysis of non-ordered categorical data. As could be demonstrated, these models bear some interesting insights into method-bias, distinguishability of different categories, as well as convergent and discriminant validity. We defined: (i) the Convergence Coefficient (*CO*; agreement rates), (ii) the Method Bias I coefficient (*MB*1; differences in latent univariate distributions), (iii) the Method Bias II coefficient (*MB*2; differences in rater-specific bivariate distributions from the average bivariate distribution), and (iv) the Distinguishability Coefficient (*Dist*; deviation of bivariate associations across raters from the expectations given the independence model). Coefficients reflecting convergent and discriminant validity for dimensional constructs generally do not reflect if agreement is higher or lower for high or low scores on the latent construct, but provide an overall estimation (e.g., consistency coefficients as amount of explained variance or Pearson correlations as measures reflecting discriminant validity). The model and coefficients presented in this contribution allow for a category-specific analysis, that is a much deeper insight into rater agreement and disagreement inspecting the specific constellations of latent categories. For example, researchers can determine if agreement is high for individuals possessing a specific property (e.g., agreement about neuroticism may be high for neurotic individuals but not for not neurotic individuals).

As the model with two-variable effects revealed, there is a considerable overall agreement rate showing that, in about 1 out of 4 cases, self- and peer raters agree with respect to neuroticism and conscientiousness. Inspecting the expected proportions may lead to the conclusion that agreement is highest for cell combinations of highly conscientious and moderately and highly neurotic individuals. The partial agreement rates also show that self- and peer raters agree more often for individuals classified in one of the above mentioned categories. Since the MTMM LCM model with two-variable interactions is a hierarchical model it “reproduces” the latent bivariate joint distributions allowing for a direct interpretation of the expected bivariate frequencies and the latent one-variable marginals. The method bias type I reveals if the latent marginal distributions differ from each other. This is not the case for neuroticism but for conscientiousness. Peers overestimate the conscientiousness with respect to the self-ratings. The category-specific agreement rates can be calculated to identify the overrepresentation in the cells on the (agreement) main diagonals. There is agreement for all cells on the two bivariate main diagonals (for neuroticism and conscientiousness).

The distinguishability index reveals in a similar manner as the category-specific agreement rates if disagreement cells are over- or underrepresented. This index can be used to detect sources of disagreement. For conscientiousness this index revealed, for example, that self- and peer raters confound the first two categories (lack of distinguishability). All other categories can be relatively well distinguished from each other for the two constructs (except for the combination of moderately neurotic in the self-report and neurotic in the peer report, which has an expected proportion as predicted by chance). This finding (if replicated and soundly estimated) might serve as a starting point to investigate the decision making process concerning these categories in more depth.

The cross-classification of latent variables belonging to the same method (heterotrait-monomethod associations) showed that there are virtually no associations for the self-report. However, the peer ratings were associated to some degree revealing that their view about personality types (combinations of latent categories) differs from the self-raters' views. Comparing these associations (for both raters) to the average association of the across raters (heterotrait-heteromethod) association yields the method bias type II. This index shows that self- and peer raters differ with respect to the latent categories. If the self-rating is considered to be a better approximation of the “true-scores” on the two latent variables a comparison of the peer reported classifications to the self-rated classification could be used as method bias type II index.

However, it is important to note, that these analyses are carried out by inspecting the table of expected proportions (relative frequencies). Deeper insights could be gained by an inspection of log-linear parameters which identify the underlying effects of the different expected frequencies. Boundary values lead to numerical problems in the computation of the parameters' variance-covariance matrix and to meaningless confidence intervals and significance tests (Galindo-Garre and Vermunt, 2005). If there are boundary values, the inverse of the information matrix cannot be determined and thus no standard errors can be calculated. Model probabilities still can be interpreted if boundary values have been found, yet, log-linear and effect-parameters are not defined (dividing by zero is not defined). Boundary values can be produced due to sparse expected frequency tables, hence, it is difficult to give recommendations for required sample sizes as the sparseness of a frequency table depends on the number of observations on the one hand but on the distribution of the observations on the other hand. With respect to the presented application, the number of boundary values points to the fact that more observations (a larger sample size) would lead to a better estimation of the model parameters.

### Extensions and future research directions

In the empirical application, the number of latent categories did not differ between the two raters and, moreover, the conditional response probabilities led to a similar interpretation of the latent classes for the two raters. This does not necessarily have to be the case in other applications. If the conditional response probabilities are not the same or comparable for the administered methods, it is not meaningful to interpret the presented coefficients in terms of agreement, yet, the analysis of the latent multivariate distribution still can give insight into the associations of the ratings. If the number of latent categories differs between the raters, but the meaning of the latent categories of the rater with fewer categories corresponds to the meaning of the other rater's latent categories, it is not meaningful to interpret overall agreement rates, but category specific agreements can still be interpreted. Moreover, the distinguishability index may be conceived as especially informative as it may reveal which of the latent categories produced by the rater with the larger number of categories cannot be separated by the other rater (if the ratings fall into the same latent category) or if there is a specific subset of categories the other rater chooses for these cases.

One possible extension of the models presented here is to incorporate the rater agreement structures presented in the first part of the contribution in the larger model for more constructs. Moreover, one might want to test if raters are interchangeable. In this case, all parameters of two peer raters sharing the same indices have to be set equal to each other (the measurement models, the latent class proportions, and all interaction terms). Nussbeck (2009) provides the necessary restrictions.

The models presented here can be directly related to the realistic accuracy model (RAM) presented by Funder (1995) and to models of signal detection theory (e.g., Wickens, 2002). The RAM provides a logic chain of determinants of accurate judgment. The knowledge about the properties of *good judges, good indicators, good targets*, and *good traits* as factors enhancing rater agreement may help to improve the quality of ratings or may help to explain why some ratings are inaccurate. Signal detection theory links the perceivable cues (visual, auditory, haptic, and olfactory) to a then activated category and to the mental registration. Analyzing these processes may be very helpful to explain how judges make up their minds depending on the cues they can perceive or the cues they even did not perceive concerning several items as “being moody,” “self-doubtful,” “sensitive,” or “vulnerable.” Integrating elements of signal detection theory, multimethod latent class models, and rater accuracy models could lead to a far better understanding of rating processes.

The major limitation of the presented models is their computational complexity. To date no software package allows for a sound estimation of the log-linear parameters of the most complex MTMM LCM models. For example, at the level of observed but also latent variables, low cell frequencies lead to estimation problems. One possible solution could be to collapse categories based on theoretical considerations (e.g., “fairly” and “very much so,” but not to collapse “not at all” and “very much so”). Yet, this would result in a loss of information and should only be considered as a first practical solution until more sophisticated solutions are presented. Hence, future research directions concern the development of better estimation procedures for the log-linear models with latent variables (e.g., Galindo-Garre and Vermunt, 2006). If these are available the applicability of the MTMM LCM in terms of required sample sizes but also in terms of properties of the estimation algorithms might be more deeply examined in simulation studies relying on empirical and/or simulated data sets. Software packages that could be used to analyze the proposed models should integrate several components: (i) better estimation procedures as Bayesian estimation methods using prior information (Maris, 1999; Vermunt and Magidson, 2005) as implemented in WinBUGS (Lunn et al., 2000) or Latent GOLD (Vermunt and Magidson, 2005), (ii) an automatic identification check as implemented in PANMARK, for example (van de Pol et al., 1996), and (iii) the possibility to run bootstrap analysis.

Future research should be conducted on analyzing large data sets which may be found in organizational psychology where many clients rate many employees. Consider a call-center where clients are oftentimes asked to rate some properties of the agent. A fixed number of clients could be randomly drawn for each agent and their agreement and disagreement as well as the convergent and discriminant validity of the evaluation scale could be analyzed. The more complex situation with differing numbers of clients for the agents could be solved adopting the multilevel-latent class approach introduced by Vermunt (2003, 2005, 2008).

Young physicians could be trained relying on the latent rater agreement models or on the multimethod latent class models if they were asked to rate patients during the ward rounds. Their ratings could be compared with ratings of other young physicians on the same patients or with the ratings of the physician in charge. This information could be used to develop specific programs to train the accuracy of the young physicians.

Data sets containing missingness on observed data which are likely to occur in many applications will additionally increase the complexity of the estimation process. Vermunt (1996) proposed an approach to analyze models with unobserved (latent), partly observed, and observed data. In this approach, response indicators have to be used indicating the missingness. This results in additional model complexity and has not yet been defined for the models presented here.

If the proposed model should proof to be applicable to empirical data situations the newly defined indices (method bias type II and distinguishability index) should be investigated in more detail. It should be examined if there is any meaningful benchmark or threshold as to which ratio is of substantial interest for given research domains. In settings with many raters, careful considerations as to which raters may provide bias free associations are necessary and will afflict the definition of this index.

The quasi-independence models offer interesting possibilities to model disagreement and agreement. If the proposed model may be soundly estimated it may also become meaningful to investigate the structure of agreement restricting the monotrait-heteromethod associations in a larger model. In the same vein, the structure of rater agreement might be adapted to three- and four-variable effects yielding non-hierarchical higher-order rater agreement models. In these models, all effects may be removed from the saturated model that do not relate to a simple, partial, or conditional overall agreement. This model would imply random associations for complete disagreement cells and might give important insights into rater bias. The clear psychometric definition and the interpretation of the log-linear parameters will be tedious because these kinds of models are no longer hierarchical. However, it might be adequate for rather distinct raters who might be expected to agree more often on some of the constructs but not to show related joint ratings for other constructs.

## Funding

This research was supported by grant Ei 379/5-1 from the Deutsche Forschungsgemeinschaft (DFG) awarded to ME. We acknowledge support for the Article Processing Charge by the Deutsche Forschungsgemeinschaft and the Open Access Publication Fund of Bielefeld University.

### Conflict of interest statement

The authors declare that the research was conducted in the absence of any commercial or financial relationships that could be construed as a potential conflict of interest.
