# Supplementary material for: Multimethod latent class analysis
Source: Front Psychol. 2015 Sep 17;6:1332. doi: 10.3389/fpsyg.2015.01332 (PMC4584970; doi:10.3389/fpsyg.2015.01332)
Supplement: Supplementary file 1 [file DataSheet1.DOCX]

Appendix A

Annotated LEM-syntax for the Multitrait-Multimethod Latent Class Model

Comments appear after an asterix (*)

lat 4 * the number of latent variables has to be specified

man 16 * the number of manifest variables has to be specified

dim 3 3 3 3 3 3 3 3 3 3 3 3 3 3 3 3 3 3 3 3

* the dimensions (number of categories) of all variables have to be specified

lab SN SC AN AC E A F B G C D H M I N J O K L P

* variables have to be labeled

mod SN.SC.AN.AC {SN.SC.AN,SN.SC.AC,SN.AN.AC,SC.AN.AC}

* the hierarchical log-linear model has to be specified relying on the highest order effects

* specification of the measurement models

A|SN {SN.A} * items A through D measure SN (self-report neuroticism)

B|SN {SN.B}

C|SN {SN.C}

D|SN {SN.D}

E|SC {SC.E} * items E through H measure SC (self-report conscientiousness)

F|SC {SC.F}

G|SC {SC.G}

H|SC {SC.H}

I|AN {AN.I} * items I through L measure AN (peer rated neuroticism)

J|AN {AN.J}

K|AN {AN.K}

L|AN {AN.L}

M|AC {AC.M} * items M through P measure AC (peer rated conscientiousness)

N|AC {AC.N}

O|AC {AC.O}

P|AC {AC.P}

rec 478 * the number of observed units has to be specified

* for ease of computation starting values (conditional probabilities) for the measurement models have been provided

sta A|SN [.2979 .4322 .2699 .0388 .0985 .8627 .0100 .0400 .9500]

sta B|SN [.4995 .4563 .0443 .0313 .1086 .8601 .0100 .0249 .9751]

sta C|SN [.6592 .1989 .1419 .4500 .5400 .0100 .0100 .1183 .8717]

sta D|SN [.5141 .3087 .1773 .2430 .1562 .6007 .0938 .1328 .7734]

sta E|SC [.7609 .2010 .0381 .0242 .7760 .1998 .0145 .0535 .9320]

sta F|SC [.8798 .1061 .0141 .0970 .8094 .0936 .0100 .0487 .9413]

sta G|SC [.2133 .3324 .4543 .0169 .2694 .7137 .0103 .0494 .9403]

sta H|SC [.7100 .2444 .0456 .1916 .5946 .2138 .0469 .1094 .8437]

sta I|AN [.6683 .3217 .0100 .0100 .4771 .5129 .0275 .0100 .9625]

sta J|AN [.6403 .3154 .0443 .1556 .5650 .2794 .0373 .0100 .9527]

sta K|AN [.7166 .1945 .0889 .6467 .2459 .1075 .3452 .2823 .3725]

sta L|AN [.7386 .1656 .0958 .4751 .3135 .2114 .2342 .2791 .4867]

sta M|AC [.8382 .1518 .0100 .0671 .7230 .2099 .0100 .0350 .9501]

sta N|AC [.9158 .0629 .0212 .0837 .7935 .1228 .0100 .0435 .9465]

sta O|AC [.4795 .3407 .1798 .0862 .3956 .5183 .0116 .0697 .9187]

sta P|AC [.8513 .1246 .0241 .2549 .6194 .1258 .0193 .1578 .8230]

dat necosepa.dat * specification of the data set

ite 100000 * maximum number of iterations for model convergence
